# Supplementary material for: Structure of the Polysaccharide Secreted by Vibrio alginolyticus CNCM I-5035 (Epidermist 4.0TM)
Source: Mar Drugs. 2020 Oct 9;18(10):509. doi: 10.3390/md18100509 (PMC7600630; doi:10.3390/md18100509)
Supplement: Supplementary file 1 [file marinedrugs-18-00509-s001.pdf]

Supplementary Figure S1. Composition analysis of the *Vibrio alginolyticus* exopolysaccharide. **A**) Gaz chromatogram of the monosaccharide composition of the polysaccharide and **B**) Gaz chromatogram of monosaccahrides standard. *: indicates peaks which couldn't be attributed with available monosaccharide standard.

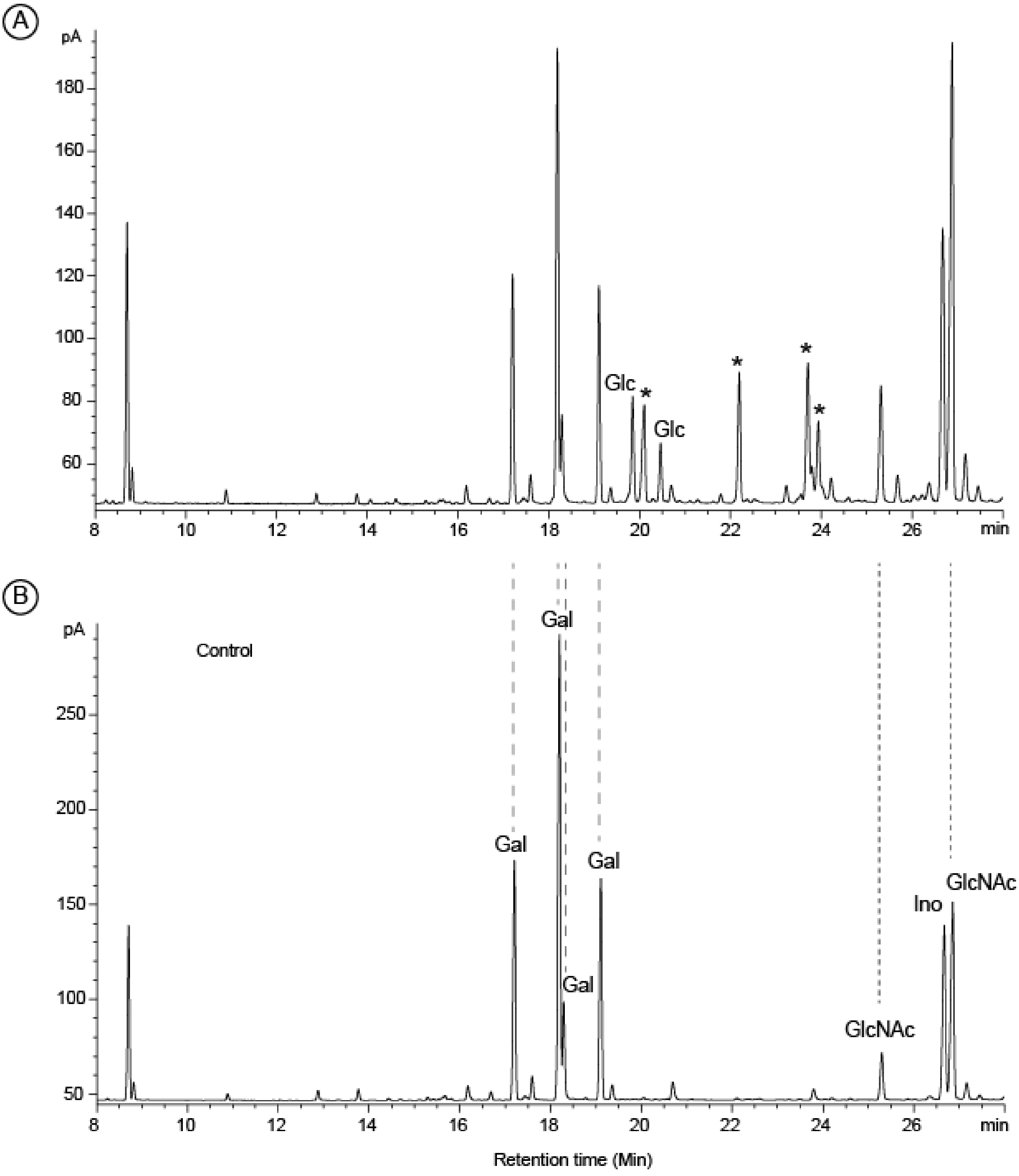

Supplementary Figure S2. Determination of absolute configuration. A) Gaz chromatogram of the trimethylsilyl-butylglycosides obtained from the hydrolysed exopolysaccharide. B) and C) D-GlcNAc and D-Gal series used as reference.

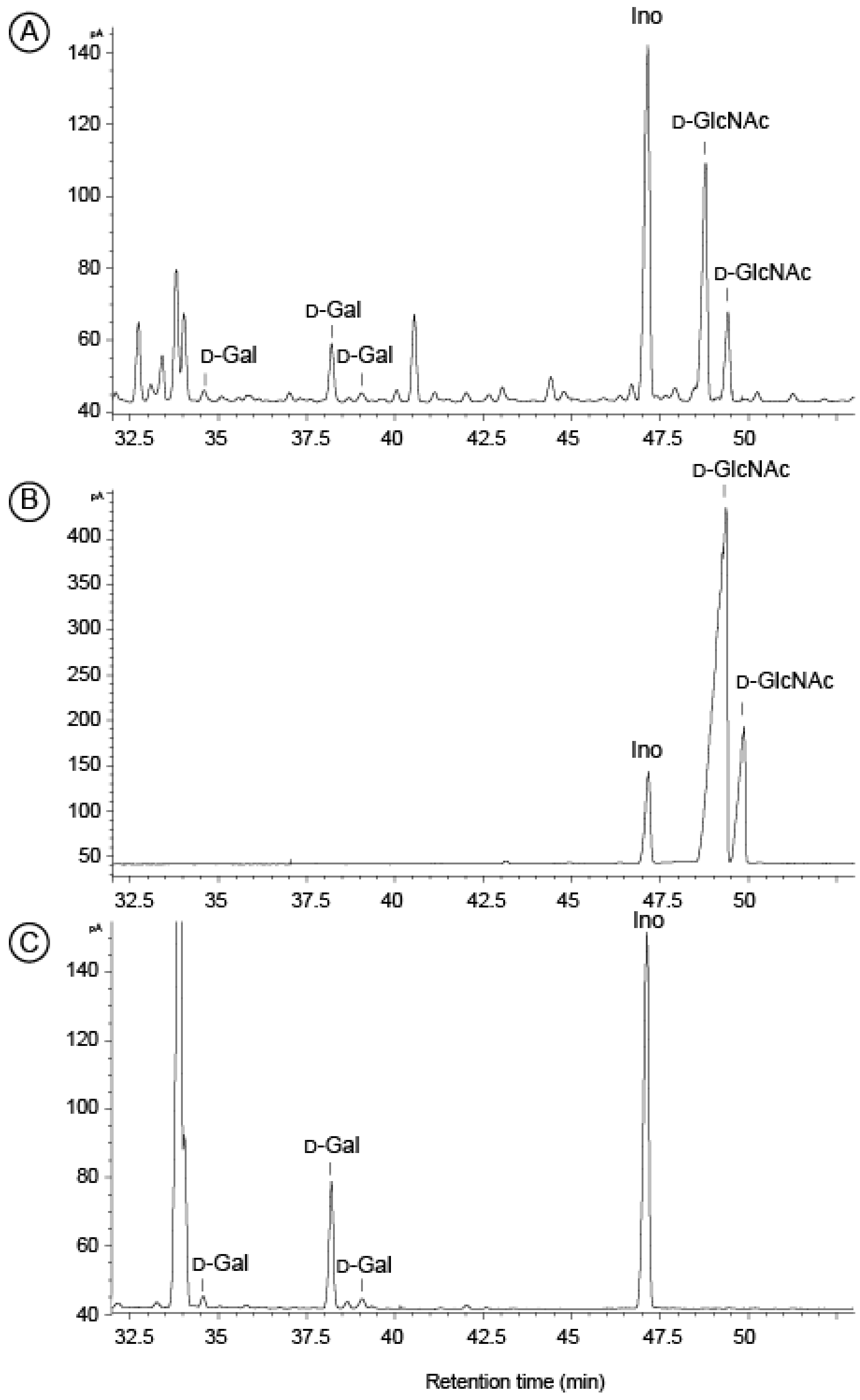

Supplementary Figure S3. COSY spectra of the *Vibrio alginolyticus* exopolysaccharide before (A) and after (B) alkaline treatment.

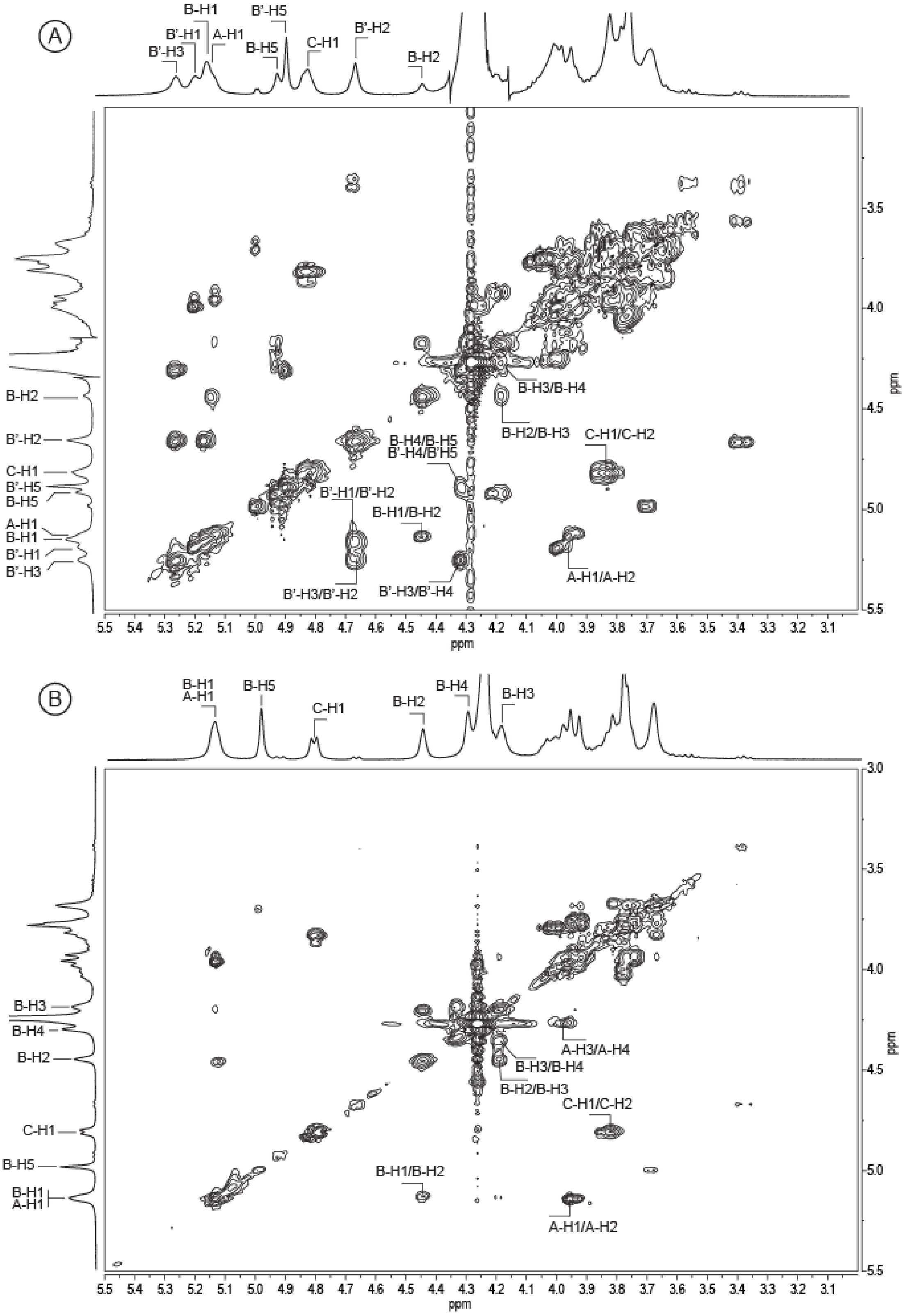

Supplementary Figure S4. HSQC spectra of the *Vibrio alginolyticus* exopolysaccharide before (A) and after (B) alkaline treatment.

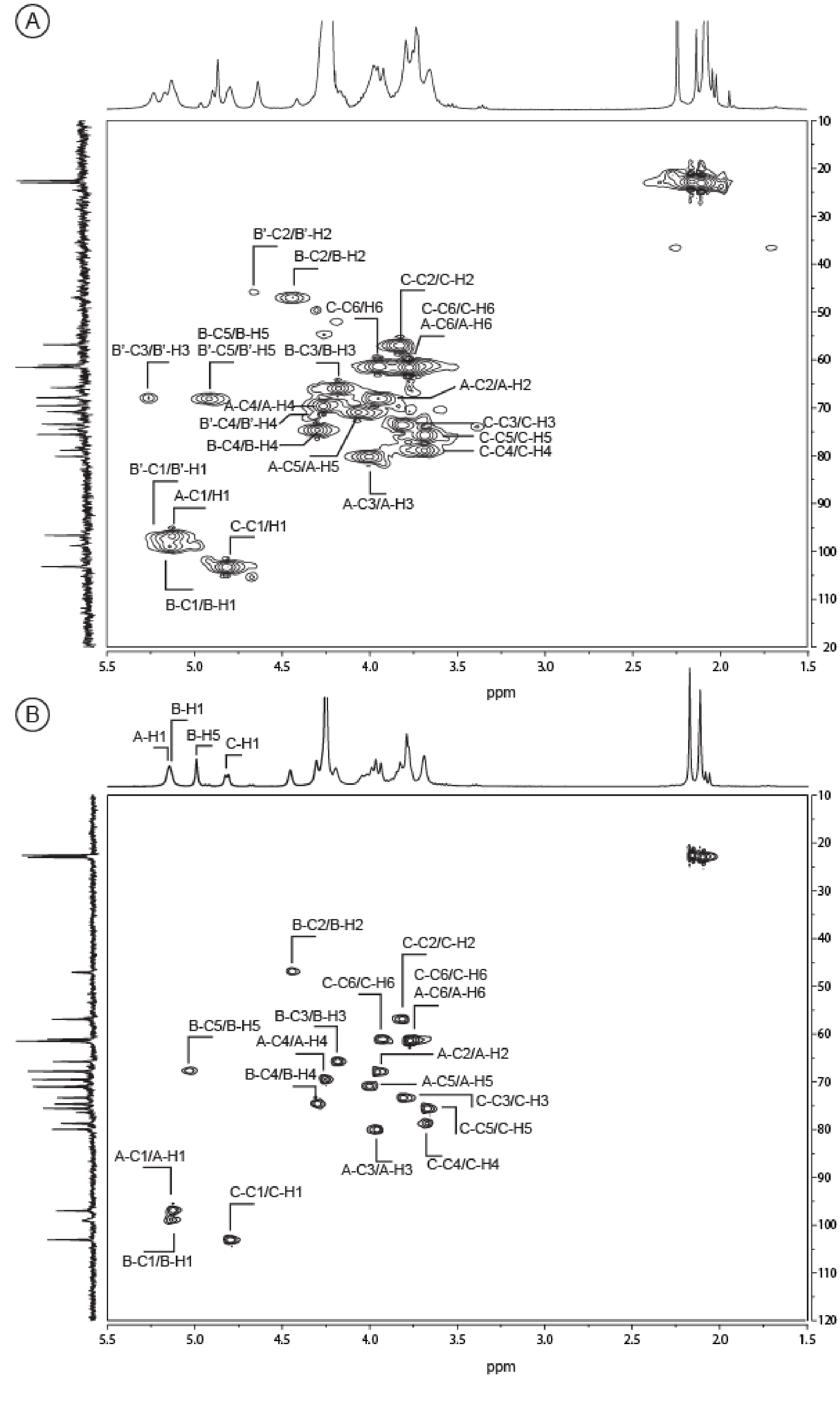

Supplementary Figure S5. COSY spectra recorded at 293K of the two disccharides purified by chromatography.

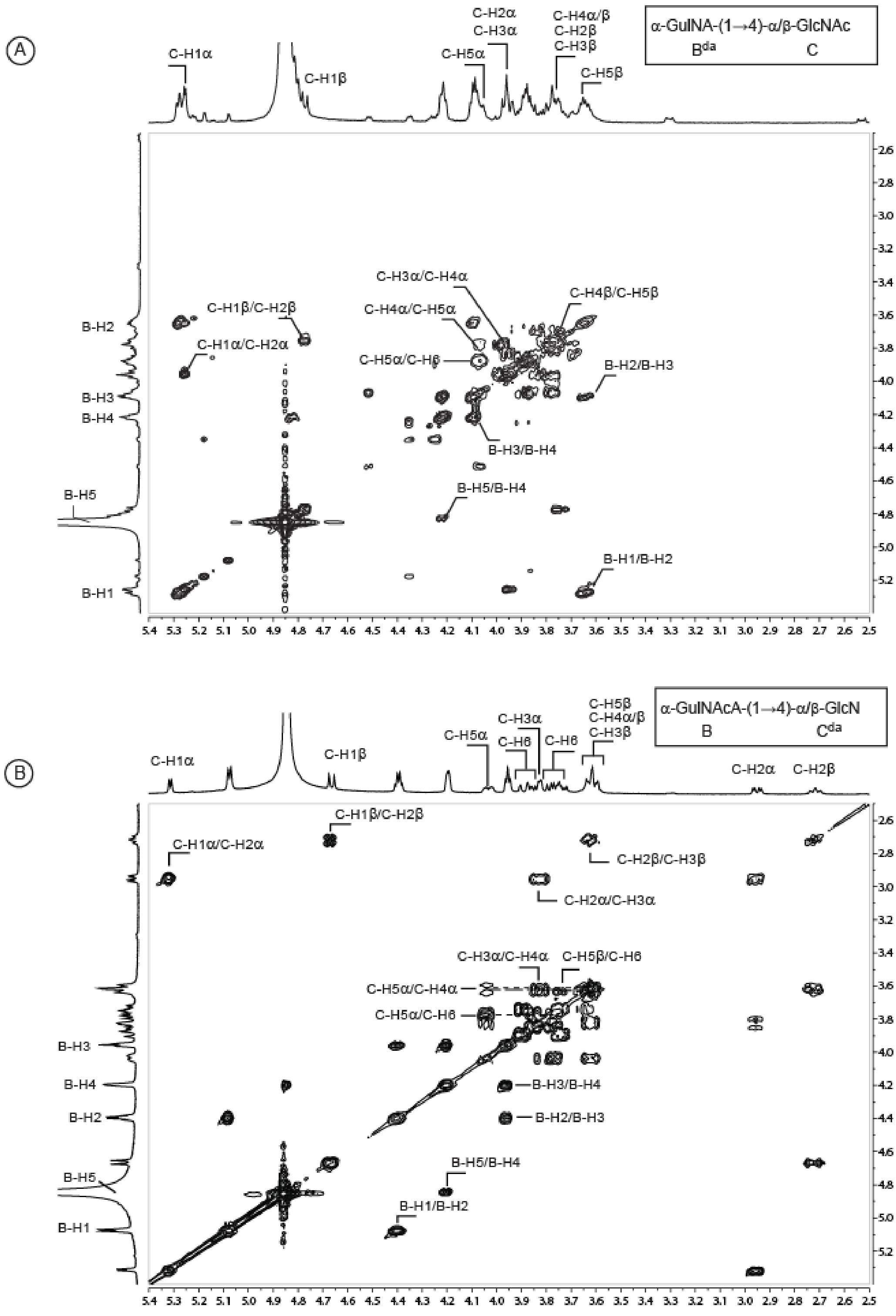

Supplementary Figure S6. HSQC spectra recorded at 293K of the two disccharides purified by chromatography.

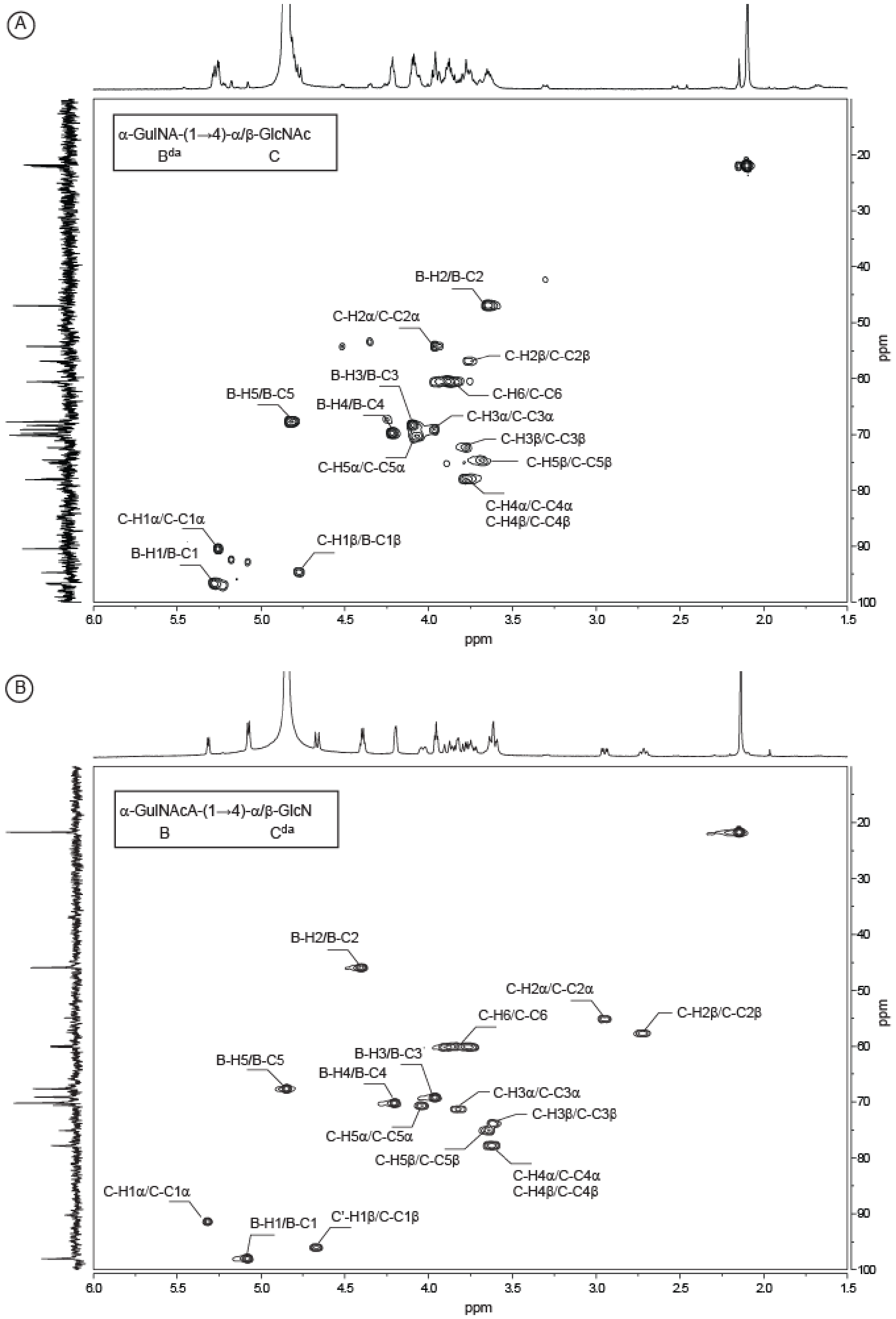

Supplementary Figure S7: Positive-ion reflectron MALDI-TOF mass spectrometry analysis of the enriched fraction of the trisaccharide obtained after hydrolysis of the *Vibrio alginolyticus* exopolysaccharide. The experiment was conducted with a MALDI-TOF/TOF Autoflex Speed apparatus (Bruker Daltonics) with 2,5-dihydroxybenzoic acid (DHB) as a matrix.

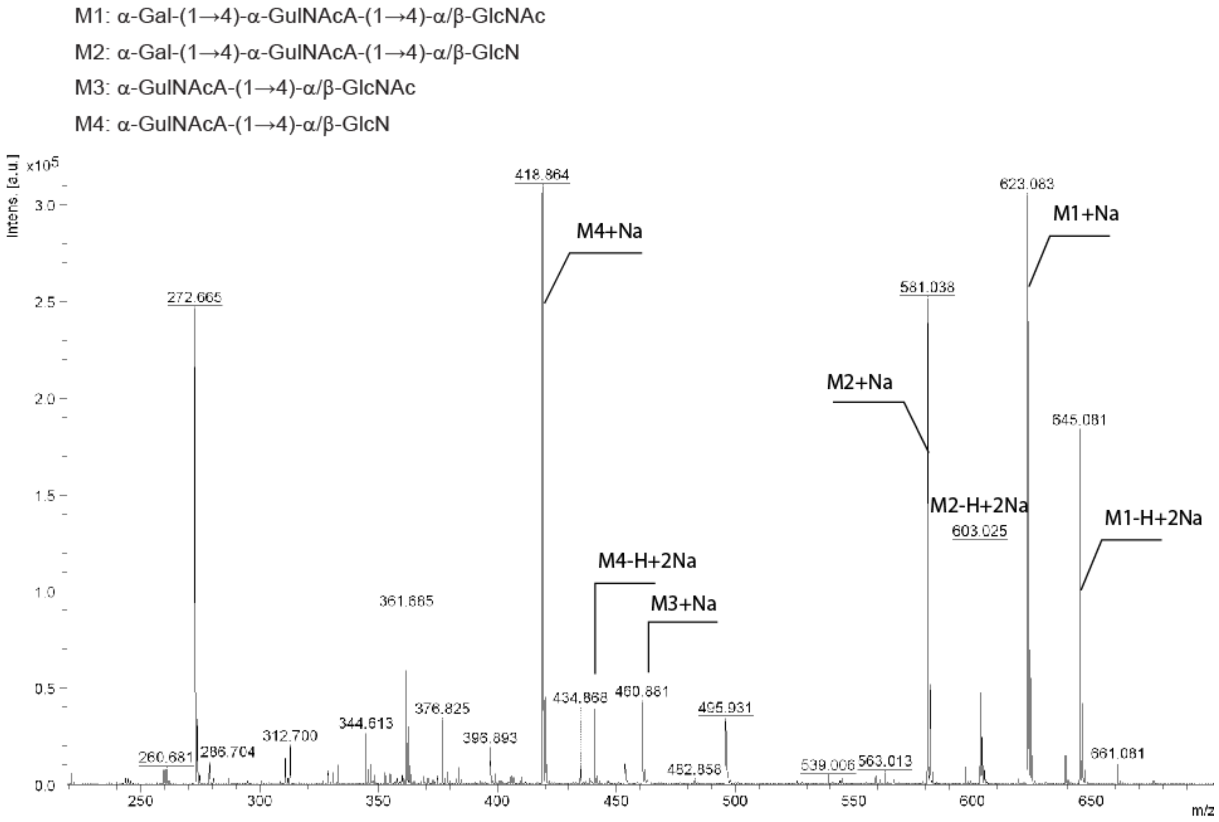

Supplementary Table 1. Comparison of the ^{13}C NMR chemical shifts (δ, ppm) of the *Vibrio alginolyticus* exopolysaccharide and the calculated values [Lipkind, *et al*. (1988) Carbohydr. Res. 175, 59-75], [a]: values of L-GulNAc [Michon, *et al*. (1985) Biochem 24, 5592], [b]: approximation using GalNAc instead of GulNAc

Sugar residue	1	2	3	4	5	6
**α-D-Gal**						
Free α-D-Gal	93.5	69.6	70.4	70.6	71.7	62.4
Calculated **α-D-Gal**-(1→4)-D-GulNAcA	101.5 [b]	68.4 [b]	80.2	70.3	72.2 [b]	62.4
Calculated **α-D-Gal**-(1→4)-L-GulNAcA	100.1[b]	68.4 [b]	80.2	70.3	72.2 [b]	62.4
Experimental	96.66	67.62	80.19	69.58	70.82	61.58
**α-L-GulNAcA**						
Free α-L-GulNAc [a]	92.4	47.5	70.4	69.6	67.1	62.2
Calculated **α-D-GulNAcA**-(1→4)-D-GlcNAc	100.0	46.5	70.2 [b]	78.9 [b]	67.3 [b]	
Calculated **α-L-GulNAcA**-(1→4)-D-GlcNAc	98.8	46.5	70.2 [b]	76.0 [b]	66.7 [b]	
Experimental	98.83	46.99	65.79	74.49	67.92	175.40
**β-D-GlcNAc**						
Free β-D-GlcNAc	96.2	58.0	75.1	71.2	77.2	62.1
Calculated α-D-GulNAcA-(1→4)-**β-D-GlcNAc**	104.0	58.0	75.4	78.6	76.4	62.1
Calculated α-L-GulNAcA-(1→4)-**β-D-GlcNAc**	104.0	58.0	73.8	78.7	77.1	61.5
Experimental	103.19	56.84	73.45	78.89	75.58	61.13
